# The clinical and operational consequences of prolonged occupancy strain and the use of non-intensive care beds to deliver critical care in a central london teaching hospital

**DOI:** 10.1186/2197-425X-3-S1-A477

**Published:** 2015-10-01

**Authors:** PA Hopkins, H Little, S Ward, C Harrington, A Feehan, K Peters, A Mcloone, M Dowling, T Chang, C Bell

**Affiliations:** 1 King's College Hospital, King's Critical Care Units, London, United Kingdom; 2 Medical Associated Software House, London, United Kingdom

## Introduction

Since 2000, there has been a marked reduction in non-clinical critical care transfers in England. ICNARC have reported that this has been associated with a reduction in case-mix adjusted mortality (1). One consequence has been the need to open critical care beds in non-specified locations in hospitals such as post anaesthesia recovery and high dependency areas.

Here we report the consequences of an unprecedented strain on ICU capacity sustained at a central London teaching hospital over a 12 month period in 2013 (graph 1). During this period, ICU capacity reached >150% requiring a number of different emergent solutions including the use of main theatre recovery and high dependency areas. We also describe some of the clinical and operational lessons we learnt.

## Objectives

Analyse the clinical and operational consequences of a sustained period of ICU occupancy in excess of 150% and describe translatable lessons learnt.

## Methods

Retrospective extraction of data from *Medtrack* ICU database (Medical Associated Software House), adverse event and microbiology databases was performed and compared with contemporaneous casemix program data. Approvals were obtained from local research ethics committees and ICNARC, maintaining subject anonymity throughout.

## Results

Standardised mortality ratio (SMR) and readmission and ICU-acquired infection rates did not change during the strain period. Interestingly, rates of *C. Difficile* which we have previously noted to increase during high occupancy, did not change. Key lessons included developing a check-list for opening unspecified beds (particularly in relation to equipment); early consideration of factors like patient dignity; maximising co-location of critical care beds; allocation of experienced nursing staff and specific medical staff to remote areas; strict rules about the category of patient that could be allocated un-specified beds and avoidance of unplanned or premature discharge from the whole critical care bed pool. Importantly, the operational responses confounded the ICNARC dataset with an apparent reduction in delayed discharge and out-of-hours discharge. The effects of strain (delirium rates in patients or burn-out in staff)-large numbers of internal bed movements; working or being cared for in non-specified ICU areas were not studied

## Conclusions

A sustained period of extreme capacity strain was not associated with any change in case-mix adjusted standardised mortality ratio or hospital-acquired infection/re-admission rates. Non-clinical inter-hospital transfers were avoided. A health economic analysis is now being conducted on these data. We believe our inter-professional critical care team deserve great credit for maintaining performance throughout this period of unprecedented operational strain.

**Figure 1:**
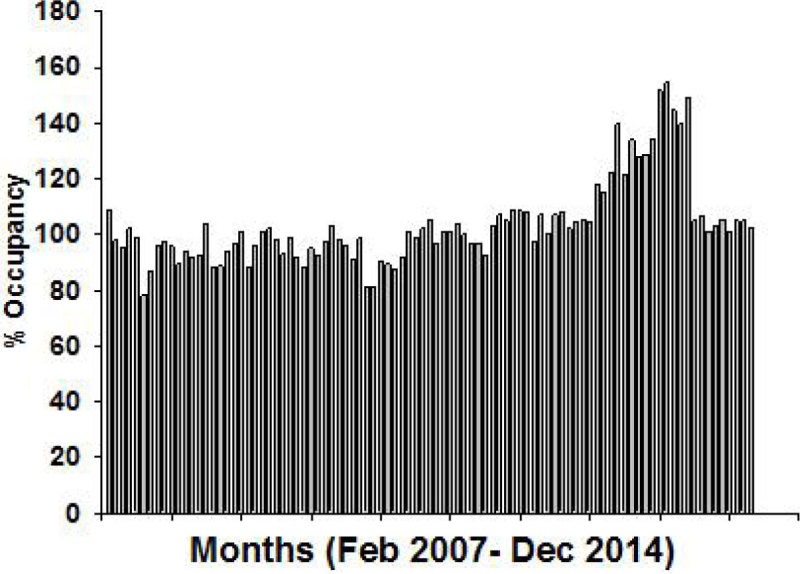
***2013 Occupancy Strain.***

## References

[1] Hutchings A, Durand MA, Grieve R, Harrison D, Rowan K, Green J (2009). Evaluation of modernisation of adult critical care services in England: time series and cost effectiveness analysis. BMJ.

